# A framework to assess the quality and impact of bioinformatics training across ELIXIR

**DOI:** 10.1371/journal.pcbi.1007976

**Published:** 2020-07-23

**Authors:** Kim T. Gurwitz, Prakash Singh Gaur, Louisa J. Bellis, Lee Larcombe, Eva Alloza, Balint Laszlo Balint, Alexander Botzki, Jure Dimec, Victoria Dominguez del Angel, Pedro L. Fernandes, Eija Korpelainen, Roland Krause, Mateusz Kuzak, Loredana Le Pera, Brane Leskošek, Jessica M. Lindvall, Diana Marek, Paula A. Martinez, Tuur Muyldermans, Ståle Nygård, Patricia M. Palagi, Hedi Peterson, Fotis Psomopoulos, Vojtech Spiwok, Celia W. G. van Gelder, Allegra Via, Marko Vidak, Daniel Wibberg, Sarah L. Morgan, Gabriella Rustici

**Affiliations:** 1 Department of Genetics, University of Cambridge, Cambridge, United Kingdom; 2 EMBL-EBI, Wellcome Genome Campus, Hinxton, Cambridge, United Kingdom; 3 MRC Human Genetics Unit, The Institute of Genetics and Molecular Medicine, University of Edinburgh, Edinburgh, United Kingdom; 4 Barcelona Supercomputing Center (BSC), INB Coordination node, Life Sciences Department, Barcelona, Spain; 5 University of Debrecen, Medical Faculty, Department of Biochemistry and Molecular Biology, Debrecen, Hungary; 6 VIB Flanders Institute for Biotechnology, VIB Bioinformatics Core, Ghent, Belgium; 7 Faculty of Medicine, Institute for Biostatistics and Medical Informatics (IBMI), University of Ljubljana, Ljubljana, Slovenia; 8 IFB-URGI, Université Paris-Saclay, Centre de Recherche INRA, Versailles, France; 9 Instituto Gulbenkian de Ciência, Oeiras, Portugal; 10 CSC - IT Center for Science Ltd, Espoo, Finland; 11 Luxembourg Centre for Systems Biomedicine, University of Luxembourg, Esch-sur-Alzette, Luxembourg; 12 DTL Dutch Techcentre for Life Sciences, Utrecht, the Netherlands; 13 Institute of Biomembranes, Bioenergetics and Molecular Biotechnologies (IBIOM), National Research Council of Italy (CNR), Bari, Italy; 14 National Bioinformatics Infrastructure Sweden (NBIS), Science for Life Laboratory, Department of Biochemistry and Biophysics, Stockholm University, Stockholm, Sweden; 15 SIB Training, SIB Swiss Institute of Bioinformatics, Lausanne, Switzerland; 16 Department of Informatics, University of Oslo, Oslo, Norway; 17 Institute of Computer Science, University of Tartu, Tartu, Estonia; 18 Institute of Applied Biosciences (INAB), Center for Research and Technology Hellas (CERTH), Thessaloniki, Greece; 19 Department of Biochemistry and Microbiology, University of Chemistry and Technology, Prague, Czech Republic; 20 Institute of Molecular Biology and Pathology (IBPM), National Research Council of Italy (CNR), Rome, Italy; 21 Genome Research of Industrial Microorganisms, Center for Biotechnology, Bielefeld University, Bielefeld, Germany; University of Toronto, CANADA

## Abstract

ELIXIR is a pan-European intergovernmental organisation for life science that aims to coordinate bioinformatics resources in a single infrastructure across Europe; bioinformatics training is central to its strategy, which aims to develop a training community that spans all ELIXIR member states. In an evidence-based approach for strengthening bioinformatics training programmes across Europe, the ELIXIR Training Platform, led by the ELIXIR EXCELERATE Quality and Impact Assessment Subtask in collaboration with the ELIXIR Training Coordinators Group, has implemented an assessment strategy to measure quality and impact of its entire training portfolio. Here, we present ELIXIR’s framework for assessing training quality and impact, which includes the following: specifying assessment aims, determining what data to collect in order to address these aims, and our strategy for centralised data collection to allow for ELIXIR-wide analyses. In addition, we present an overview of the ELIXIR training data collected over the past 4 years. We highlight the importance of a coordinated and consistent data collection approach and the relevance of defining specific metrics and answer scales for consortium-wide analyses as well as for comparison of data across iterations of the same course.

This is a *PLOS Computational Biology* Education paper.

## Introduction

The ELIXIR Training Platform aims to ‘strengthen national [bioinformatics] training programmes, grow bioinformatics capacity and competence across Europe, and empower researchers to use ELIXIR’s services and tools’ (https://elixir-europe.org/platforms/training). To this end, the ELIXIR EXCELERATE ‘Training Quality and Impact Subtask’, in collaboration with the ELIXIR Training Coordinators Group, endeavoured to collect and analyse feedback data from training participants who attended ELIXIR training events between September 2015 and August 2019 in order to (1) provide ELIXIR and its stakeholders with knowledge of the training effort, quality, and impact of its training programme and (2) make best practices available to new training providers for assessing their courses. This framed a data-driven approach to assess ELIXIR’s training quality in the short term (directly after the training had taken place) and training impact in the longer term (6 months to 1–2 years after training had taken place, in 6-month intervals).

ELIXIR training events are typically 1–5 days long and cover topics including basic programming, introduction to specialised bioinformatics pipelines and tools, data management, and instructor training. Some courses are developed under ELIXIR as a community effort, such as the ELIXIR EXCELERATE Train-the-Trainer (TtT) programme [1,2], the Genome Assembly and Annotation programme [3], and the ELIXIR-Carpentries programme [4,5], whereas others are developed by individual ELIXIR member states (i.e., ELIXIR Nodes) to meet the training needs of their research communities. All courses typically include hands-on practical sessions, are organised around learning objectives, and are aimed at postgraduate students and researchers in the life sciences, although courses are also open to other employment sectors, such as industry, healthcare, and nonprofit organisations. Because ELIXIR training is spread across a distributed infrastructure (ELIXIR comprises 22 ELIXIR Nodes, with many institutions within each ELIXIR Node), most of the training courses vary in curriculum and in the way in which the course is developed and delivered. Further, ELIXIR brings together training providers at different levels of maturity: some run large training programmes, others run a few events each year, and others are new training providers who are just beginning to develop training. In order to address these complexities, we had to (1) uncouple curriculum design, to a certain extent, from measuring training quality and training impact by ensuring that the quality and impact assessment project aims and metrics were as general as possible and (2) ensure that data were collected in a coordinated way by engaging the ELIXIR training coordinators, who coordinate training for their respective ELIXIR Node.

By monitoring and evaluating training, one is better placed to assess its quality and make evidence-based recommendations for change, if needed, as well as to determine the impact that the training is having and whether intended targets are being met. For example, The Carpentries capture data relating to participants’ demographics, tool usage, and self-perceived confidence in working with data [6]. In the longer term, when some time has passed after training, the impact of the training on attendees’ work has been assessed by collecting data regarding, for example, research outputs, collaborations, change to practice, and career progression [7,8].

Criticism of the efficacy and impact of short-format training, such as from Feldon and colleagues [9], has been a source of interest and discussion for the community, with those advocating for short-format training arguing that the context in which training is delivered is important when determining efficacy and that the impact of short-format training may be successfully assessed by focusing on target outcomes that are conducive to self-evaluation, such as changes to practice, change in confidence, etc., as opposed to performance metrics, such as formal assessment [10]. In addition, the items measured in the Feldon and colleagues paper [9] have been criticised by members of the community because they do not necessarily correlate with the intended learning outcomes of the training programmes included in their study, which may have led to perceived underperformance [11]. Although it is acknowledged that long-format training may be preferred, especially when lots of content needs to be covered, often this type of training is impractical, not possible, and/or not available, especially in fields in which the landscape is constantly changing. Therefore, short-format training should be optimised within the limitations by focusing on clear outcomes [10].

This work outlines the coordinated, consortium-wide strategy of the ELIXIR EXCELERATE Training Quality and Impact Subtask with regard to assessment of the ELIXIR training programme. Data included in this work cover the period of 1 September 2015 to 6 August 2019.

## Defining project aims for assessing ELIXIR’s training quality and impact

In order to ensure that we collected information that was relevant to ELIXIR, that focused on target outcomes, and that could be applied to a wide range of training activities developed and organised by different training providers, we consulted with our stakeholders to determine project aims, thereby defining the scope of the project.

The overall aims of this project can be summarised as follows (detailed project aims provided in S1 File):

- describe the audience demographic being reached by ELIXIR training events,
- assess the quality of ELIXIR training events directly after they have taken place, and
- evaluate the longer-term impact that ELIXIR training events have had on the work of past participants.

By collecting information on the audience demographic, we wished to profile who had been reached by the ELIXIR training programme, whether there are audiences that are underrepresented, and whether there are unintended biases. We were interested in participant satisfaction as a reflection on training quality in order to be able to inform best practice for ELIXIR training. We acknowledge that training quality is more complex than solely participant satisfaction and that the community would benefit from future work to obtain a fuller picture on training quality. We were interested in training impact in order to examine the effect that ELIXIR training had on the work and career of past participants and the extent to which the learning had been passed on to others, in accordance with ELIXIR Training Platform’s definition of training impact (May 2018): ‘A measure of how participation in a training course improves someone’s understanding and awareness of a particular domain/topic, leading to change in their research/professional development as well as passing on of the knowledge/skills acquired to others.’

## Data collection: Metrics

In an effort to achieve the above aims, we compiled a set of core metrics and associated questions and defined answer scales for measuring audience demographics and training quality in the short term and training impact in the longer term. These metrics were developed out of those already collected by ELIXIR training providers, as well as from discussions with stakeholders, external training providers, and literature review [6,7]. A summarised list of metrics may be viewed in the Supporting information (S2 File), and the full list may be viewed at the following link: https://training-metrics-dev.elixir-europe.org/references. Although the majority of these metrics are quantitative in nature, from a best practices point of view we also advocated for including qualitative questions in order to ensure that nuanced learning experiences were captured. Further, we advocated for including comment boxes for the quantitative questions when possible. Data were collected via feedback survey following a training event. In some cases, the audience demographic information was collected via registration form prior to the training event. Further to the above metrics, training event information—such as event title, event website, number of participants, number of trainers, funding source, etc.,—was collected for all ELIXIR training events in order to contextualise the feedback data and comment on the reach of ELIXIR training (https://training-metrics-dev.elixir-europe.org/references?title=&field_reference_type_value=Event). Limitations of our approach, as well as insights regarding how to mitigate these challenges, are outlined in the Supporting information (S3 File) as a reference for others that might want to set up a similar quality and impact assessment activity.

Training providers were encouraged to use a survey tool that was accessible to them because some research institutions have requirements for specific systems and tools to be used to collect participant data. Regardless of the tool used, all data were required to be collected and stored securely, in accordance with the General Data Protection Regulation (GDPR). In the case of ELIXIR, anonymised data—collected by each training provider—were uploaded to an internal, bespoke database to allow for ELIXIR-wide analyses.

## Data collection: Strategy

**Fig 1:**
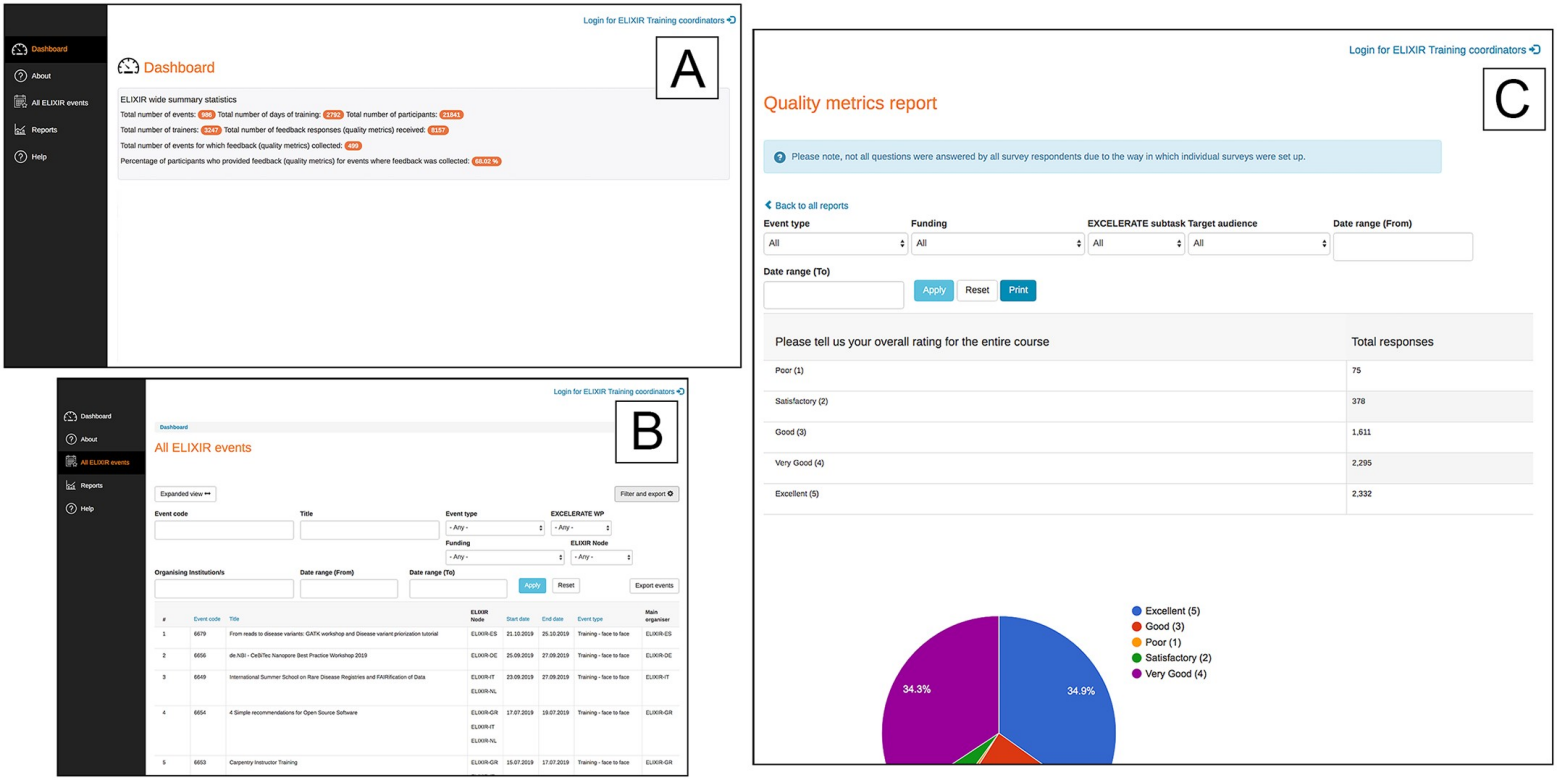
ELIXIR’s training metrics database. The database may be publicly accessed at https://training-metrics-dev.elixir-europe.org/. (A) A summary of ELIXIR-wide training may be viewed on the ‘Dashboard’. (B) A list of all ELIXIR training events may be viewed on the ‘All ELIXIR’ events page. (C) Users may generate interactive reports on the ‘Reports’ page. (Screenshots were taken on 6 August 2019).

In an effort to move away from spreadsheets and data sharing via email for the collection of training event data and feedback data from ELIXIR Nodes, we developed a bespoke training metrics database (https://training-metrics-dev.elixir-europe.org/) built on Drupal and hosted on Pantheon (Fig 1). The database architecture is based on a database previously developed at the European Bioinformatics Institute (EMBL-EBI) for collecting data pertaining to their training programme. The database does not automate data collection, per se, but rather simplifies and streamlines data collection and storage, which in turn aids the controlled access to, visualisation of, and reporting on the data. Each ELIXIR training coordinator has a unique account through which they upload and visualise data pertaining to their own ELIXIR Node, as well as visualise summaries for the overall ELIXIR training portfolio. Data may be filtered according to areas of interest (as per the training event information filters) in order to contextualise the data, and bespoke reports may be generated. The coordinated collection of specific training metrics with defined answer options has introduced the consistency needed for data analysis and generation of summaries of the ELIXIR-wide training provision. Coordinated collection has also facilitated populating the database with all data collected to date; significant effort went into consolidating the data and sourcing all missing information for all ELIXIR training events. The database was launched on 10 June 2019, and ELIXIR training coordinators are now uploading their training data directly to it. Overall training statistics for the period of 1 September 2015 to 6 August 2019 may be viewed in Table 1. It is worth mentioning that the feedback collection strategy was only implemented at the end of 2016, whereas the total number of events shown is from the beginning of EXCELERATE, which commenced in 2015. This explains why feedback was not collected for all events, because prior to implementing the strategy, it was up to each institution to collect feedback data, and this was not happening at all sites. Latest statistics may be viewed on the database ‘Dashboard’ (https://training-metrics-dev.elixir-europe.org/).

**Table 1:** ELIXIR-wide training event statistics (data accessed from the training metrics database on 6 August 2019).

**Number of training events**	986
**Number of days of training**	2,792
**Number of trainers/facilitators**	3,247
**Number of individuals trained**	21,841
**Number of feedback responses received (corresponding to the quality metrics set of data)**	8,157
**Number of events for which feedback was collected**	499
**Percentage of participants who provided feedback for events in which feedback was collected (corresponding to the quality metrics set of data)**	68%

## The quality and impact of ELIXIR training

A summary of ELIXIR’s training quality and impact data may be viewed on the ‘Reports’ page of the training metrics database (https://training-metrics-dev.elixir-europe.org/all-reports). Below, we highlight some of the metrics collected (values accessed from the training metrics database on 6 August 2019).

The majority of ELIXIR training participants are PhD candidates (44.8%) or postdoctoral researchers (26.1%) from academia/research institutions (92.9%), which is expected for ELIXIR courses because this is our main target audience. The overall gender balance of the participants is 52.7% female and 46.4% male (0.8% prefer not to say), suggesting balanced gender representation at training events. Although most training takes place in Europe, training participants represent over 60 nations, indicating that ELIXIR training has a wide reach. In total, 69.2% of survey respondents indicated that the training event they attended was ‘Excellent’ or ‘Very Good’, and 89.9% indicated that they would recommend the event to others. In total, 83.9% of survey respondents indicated that they would use the tools and/or resources covered in the training again. Overall, it is apparent that the ELIXIR training programme targets a particular audience in need of basic bioinformatics skills training, has a wide reach, and appears to be of a high quality.

**Fig 2:**
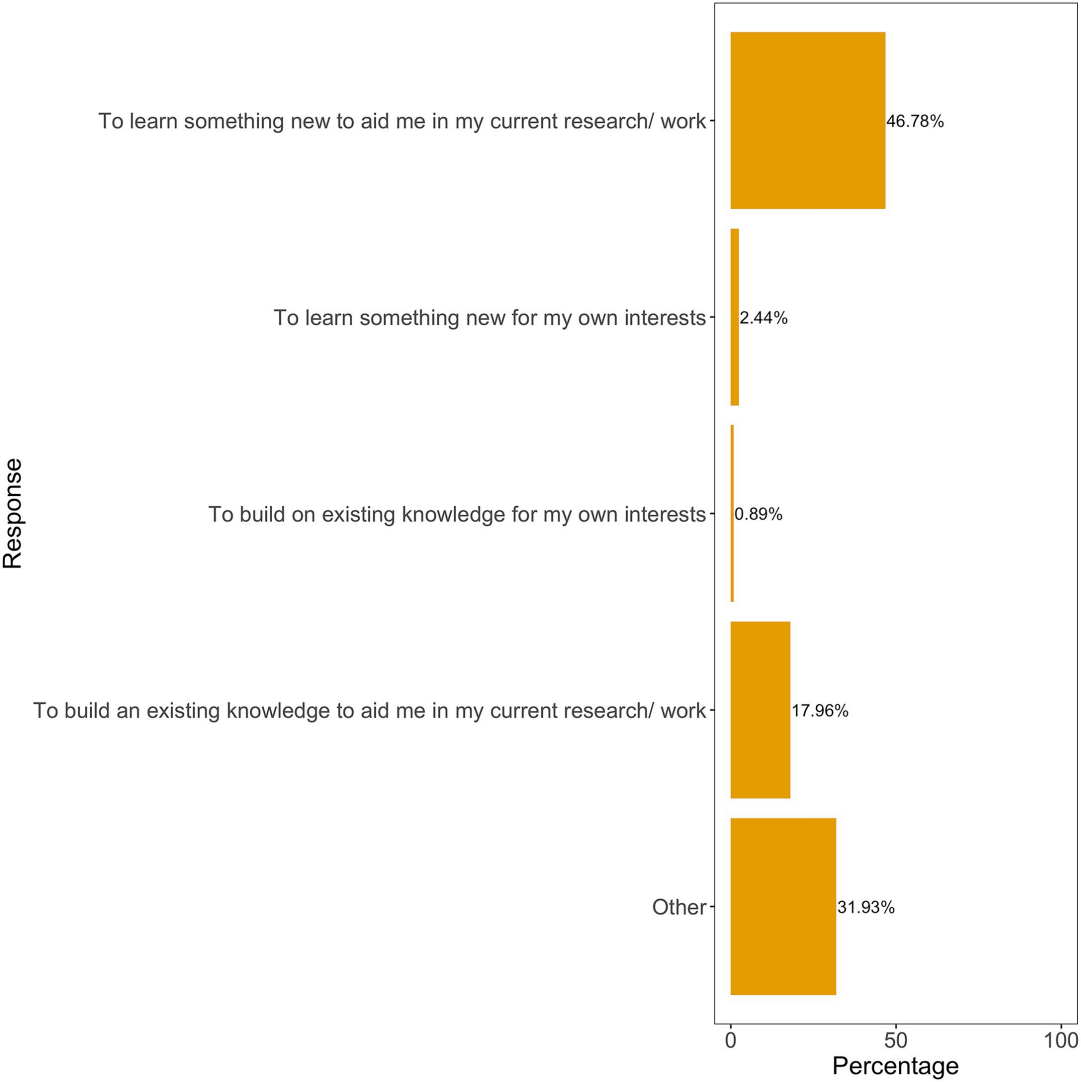
Why did you attend the training?. Past participants reflected on their reasons for attending the training. Graph generated in the R environment using the tidyverse package [12] and ggplot2 theme adapted from https://orchid00.github.io/ElixirBE/reports/2018March_nt.

A subset of individuals agreed to being contacted in the future for further feedback and subsequently responded to surveys 6 months to 1–2 years after training (Table 2; distribution of how long ago training was attended: ‘less than 6 months’—23.7%; ‘6 months to a year’—41.9%; ‘Over a year’—34.4%). Approximately 11% of participants responded, which is in line with what is generally expected for long-term feedback response rates in the field. The majority of respondents indicated that they attended the training event ‘to learn something new to aid me in my current research/work’ (46.8%) or ‘to build on existing knowledge to aid me in my current research/work’ (17.9%) (Fig 2).

**Table 2:** Long-term feedback collection (6 months to 1–2 years after training).

**Number of training events for which feedback was collected**	129
**Number of individuals trained**	2,977
**Number of feedback responses received**	328
**Percentage of participants who provided feedback for events in which long-term feedback was collected**	11%

The majority of survey respondents indicated that they use the tools and/or resources covered in the training ‘frequently (weekly to daily)’ (31.2%) or ‘occasionally (once in a while to monthly)’ (58.9%) in comparison to ‘never’ (45.8%) before having attended the training, which indicates positive uptake of the resources covered in the training. The majority of respondents indicated that they had already recommended the training to others or intended to do so (92.5%) and indicated that they had shared the training with others (60.4%).

**Fig 3:**
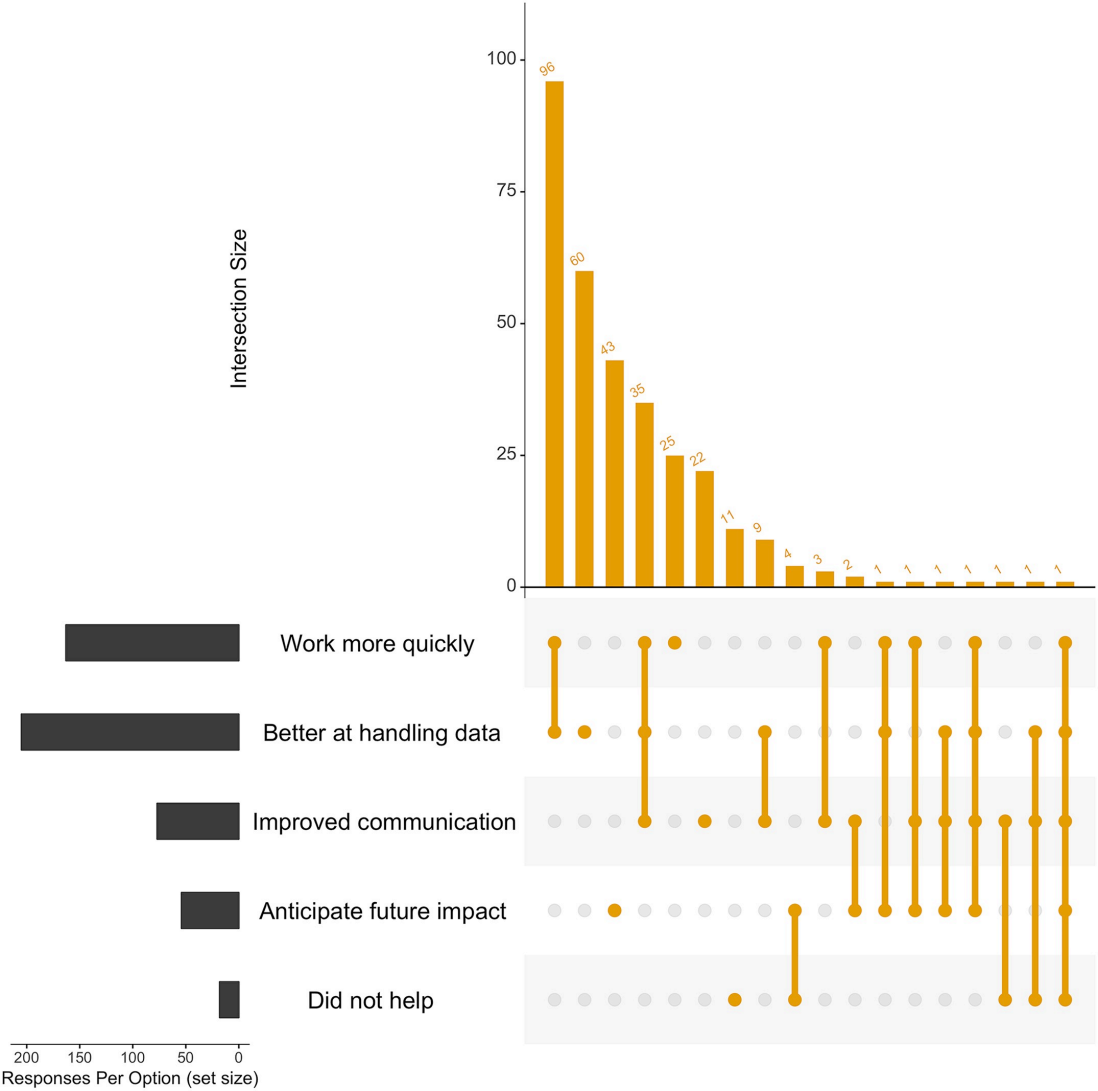
How did the training help with your work?. Each respondent was able to select multiple answers to this question. The ‘upset diagram’ illustrates how participants felt that the training has helped them with their work (‘Response Per Option (set size)’, lower LHS) as well as the different combinations of answers selected by the same individual, represented by the size of the intersection (‘Intersection Size’, RHS, ‘orange’). Packages used to create this graph (and graph in Fig 4) in the R environment were tidyverse [12] and UpsetR [13]. LHS, left-hand side; RHS, right-hand side.

**Fig 4:**
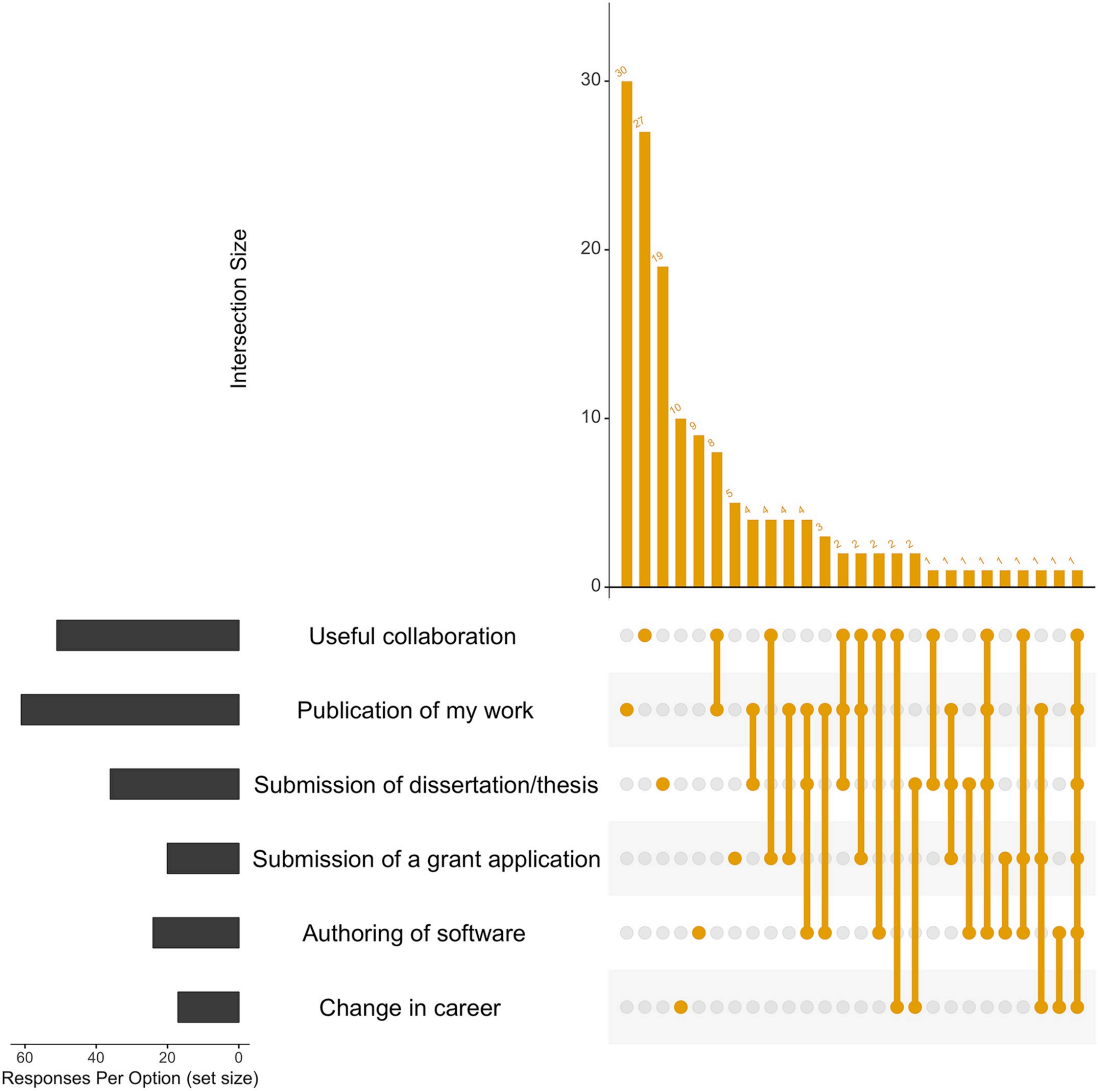
Did the training lead to or facilitate any of the following outcomes?. Each respondent was able to select multiple answers to this question. The ‘upset diagram’ illustrates what outcomes the training has led to or facilitated (‘Response Per Option (set size)’, lower LHS) as well as the different combinations of answers selected by the same individual, represented by the size of the intersection (‘Intersection Size’, RHS, ‘orange’). LHS, left-hand side; RHS, right-hand side.

From Fig 3, it is apparent that most survey respondents indicated that the training ‘improved my ability to better handle data’. Interestingly, many respondents said that the training helped both in that it ‘improved my ability to better handle data’ as well as ‘enabled me to complete certain tasks more quickly’. This co-occurrence was maintained irrespective of how long ago training was attended (Fig A-C in S4 File). From Fig 4, it is apparent that ELIXIR training has facilitated tangible outcomes, such as publication of participants’ work and useful collaborations (general trends maintained over time periods surveyed, Fig E-G in S4 File). Although roughly half of the respondents indicated that none of the outcomes listed had been achieved at the time of survey completion (Fig D in S4 File), this is consistent with the fact that a relatively short period of time had elapsed between course attendance and filling in this survey, indicating that some of these outcomes likely require a longer time frame than 1 or 2 years in order for them to be accomplished. Overall, it is apparent that the ELIXIR training programme has had a positive impact on the work of training participants.

For the TtT programme, which aims to build a network of bioinformatics trainers across Europe, we asked additional questions relating to how TtT had impacted the participants’ teaching practice since attending the training. Of the 90+ survey respondents, 70.4% had gone on to train or organise training. In total, 14.3% indicated that they had established training partnerships with other ELIXIR Nodes as the result of attending the TtT event, and 93.1% indicated that they have or intend to share the training with others. Because of the capacity-building nature of this effort, the impact is multiplied through strengthening local trainer capacity and increasing training opportunities.

## Recommendations and looking forward

We attribute the ability to comment on quality and impact metrics for ELIXIR training as a whole to our coordinated and consistent data collection approach as well as our attention to defining specific metrics and answer scales. Such an assessment framework is applicable for those coordinating quality and impact assessment of training for any discipline regardless of their setup, whether that be within a single institution or across multiple institutions, and similarly for a single recurring training event or for an entire training programme; in each case the same principle of combining quantitative data for analysis applies. Further, data collected can be used for programme or training event monitoring—for example, whether intended audiences are being reached, identifying gaps in the demographic served, tailoring of courses, identifying areas for improvement, reflecting on the effectiveness of the training—as well as for reporting on training to funders and other stakeholders. The strategy presented in this paper has been endorsed by the ELIXIR Heads of Node Committee, which has a major role in developing and approving the ELIXIR scientific and technical strategy. Further, the strategy will form part of the ELIXIR training tool kit (to be developed) and has been adopted by other European Union–funded projects, such as European Open Science Cloud (EOSC)-Life (https://www.eosc-portal.eu/eosc-life) and the European Joint Project on Rare Diseases (https://www.ejprarediseases.org/).

Looking forward, it might be interesting to collect additional data that allows us to comment on a fuller picture of training quality, such as the quality of the learning materials. In addition, it would be interesting to comment on whether participant feedback varied with workshop context—for example, are long events more diverse than short events in terms of activity types covered (e.g., instructor-led sessions, tutorial sessions, hackathon sessions), do long events allow more opportunities for networking and cohort building, etc., and what inferences may be made from this? Although the focus of this work has been on the impact of training on the individual, the next step would be to estimate the impact of training efforts on the wider scientific community (namely, the ELIXIR platforms and communities), on ELIXIR as a research infrastructure, and possibly to quantify return on investment. To ensure that the impact of ELIXIR training is factored into wider discussions on ELIXIR’s overall impact, we have been involved in conversations regarding measuring the socioeconomic impact and long-term sustainability of research infrastructures through taking part in Research Infrastructure Impact Assessment Pathways (RI-PATHS) (https://ri-paths.eu/) events, as well as through contributing to ELIXIR’s long-term sustainability planning.

In summary, we recommend the following for those wanting to assess training quality and impact:

- Define a common set of quality metrics.
- Define the impact that you hope to have and develop an impact statement accordingly.
- Define metrics and associated answer scales in order to demonstrate these measures of quality and impact—define answer options when possible to facilitate data analysis because free text is more difficult to analyse; however, when possible, we advise including free text comment boxes in addition to the defined answer options to ensure that alternate responses are not missed.
- Consolidate data collection whether across a consortium or for different courses from the same training programme or for multiple occurrences of the same training event.
- Collect training event data (e.g., course type, start and end date, etc.) in order to contextualise findings.
- Limitations may be mitigated against by careful planning, including specifying a single route for data collection, allowing for time at the end of the training for participants to fill in the survey and stressing the importance of the collected data to incentivise participants to fill it in, making provision for longer-term feedback collection in the project planning stage, and taking note of GDPR or similar restrictions when collecting and storing data.

## Supporting information

S1 File Detailed project aims. (DOCX)

S2 File Metrics. (DOCX)

S3 File Limitations of the approach. (DOCX)

S4 File Long-term feedback data supplementary figures. (DOCX)
