# Supplementary material for: A framework to assess the quality and impact of bioinformatics training across ELIXIR
Source: PLoS Comput Biol. 2020 Jul 23;16(7):e1007976. doi: 10.1371/journal.pcbi.1007976 (PMC7377377; doi:10.1371/journal.pcbi.1007976)
Supplement: S1 File — (DOCX) [file pcbi.1007976.s001.docx]

## S1 file, Detailed project aims.

For all ELIXIR training events in the short term (i.e. directly after training), including Train the Trainer (TtT) events, the project aimed to:

1. Describe audience demographic, and number of individuals, reached by ELIXIR Training events.
2. Determine the most effective dissemination channels for ELIXIR Training events.
3. Assess the potential uptake of tools/resources covered in the training as the result of attending ELIXIR Training events.
4. Assess how satisfied participants of ELIXIR Training events are and whether they would recommend the training to others.

For selected ELIXIR Nodes that have the capacity to follow up with past participants, and for selected course types (e.g. short courses of 1-5 days in length, courses with narrow range of learning outcomes etc.) the project aimed to assess the following, 6 months to one/two years after training, in 6-month intervals:

1. Whether participants who attended ELIXIR Training events in the past have increased their usage of the resources/tools covered in the training.
2. Participants’ self-rated confidence in using certain tools/resources since taking part in the training as well as how the training has helped with their work and whether this outcome aligns with participants’ subjective desired outcome.
3. Whether the training facilitated or led to new collaborations, publications, submission of degree dissertations/theses, submission of grant applications, authoring of software etc.
4. To what extent the training has been recommended and to what extent the learning has been cascaded to others.
