# Supplementary material for: A framework to assess the quality and impact of bioinformatics training across ELIXIR
Source: PLoS Comput Biol. 2020 Jul 23;16(7):e1007976. doi: 10.1371/journal.pcbi.1007976 (PMC7377377; doi:10.1371/journal.pcbi.1007976)
Supplement: S2 File — (DOCX) [file pcbi.1007976.s002.docx]

## S2 File - Metrics.

### Audience demographic and training quality metrics

To assess the audience demographic and training quality in the short term and consequently to address the short-term project aims, a set of metrics and associated questions (with defined answer scales) was developed. An overview of the audience demographic metrics and training quality metrics is provided below. A full list of the questions and answer scales may be viewed at <https://training-metrics-dev.elixir-europe.org/references>.

Audience demographic metrics:

- Participants’ career stage
- Participants’ employment sector
- Participants’ gender
- Participants’ country of employment
- Where did the participants see the course advertised?

Training quality metrics:

- Had the participants used the tool(s)/resource(s) covered in the course before?
- Will the participants use the tool(s)/resource(s) covered in the course again?
- What are the participants’ overall satisfaction with the course?
- Would the participants recommend the course?
- Are the participants willing to be contacted for further feedback in the future? (for purposes of contacting them for long term feedback, that is to say training impact assessment).

### Training impact metrics

To assess the impact of ELIXIR Training in the longer term according to the long-term project aims, a set of metrics and associated questions and defined answer scales was developed.

ELIXIR Training Platform’s definition of training impact (May 2018) is as follows:

*A measure of how participation in a training course improves someone’s* ***understanding and awareness*** *of a particular domain/topic, leading to* ***change in their research/professional development*** *as well as* ***passing on of the knowledge/skills*** *acquired to others.*

Each component of ELIXIR’s training impact definition (highlighted in bold above) was unpacked to determine metrics that may be collected to measure the respective component, which in turn gave rise to the long-term project aims. These themes are explored below. A full list of the questions and answer scales may be viewed at <https://training-metrics-dev.elixir-europe.org/references?title=&field_reference_type_value=Impact+metrics>.

**Change in understanding and awareness:** Participants’ change in awareness of a particular tool/resource, in relation to a specific topic/domain, as the result of attending ELIXIR Training. As measured by determining:

- Participants’ self-reported use of tool/resource both before attending and after attending the training.
- Participant-specified rating of confidence with a particular tool/resource.

**Change in practice (research/professional development) and tangible outcomes:** Participants’ change in ability/work/career:

- How has the training helped with the participants’ work?
- How does the participants’ initial motivation for wanting to attend a training event compare to how the training has helped with their work - i.e. did their desired outcome match the actual outcome?
- Did the training facilitate or lead to useful collaborations, publications of work, submissions of dissertation/thesis for degree purposes, submissions of a grant application, authoring of software?

**Passing on of the knowledge/skills to others:** To what extent has the learning been cascaded to others? As measured by:

- How many people have the participants’ taught the skills and/or knowledge to that was learnt during the training?
- Have the participants recommended the course to others?

In addition to the above, the following metrics were collected for ELIXIR Train-the-Trainer activities, such as the ELIXIR EXCELERATE Train the Trainer programme and the ELIXIR-Carpentries Instructor Training, in order to examine the extent to which these programmes had affected the teaching practice of attendees. These metrics also assist in addressing the long term project aims:

- Since attending the training: how many courses have the participants organised, led, or co-lead; how many of these were ELIXIR courses, and how many individuals, approximately, have been on the courses, overall?
- Have the participants established training partnerships with other ELIXIR Nodes as the result of attending the training event?
- How has the training changed the participants’ teaching practice?
