# Supplementary material for: A framework to assess the quality and impact of bioinformatics training across ELIXIR
Source: PLoS Comput Biol. 2020 Jul 23;16(7):e1007976. doi: 10.1371/journal.pcbi.1007976 (PMC7377377; doi:10.1371/journal.pcbi.1007976)
Supplement: S3 File — (DOCX) [file pcbi.1007976.s003.docx]

## S3 File, Limitations of the approach.

We feel that it is important to outline the limitations of our approach in order to assist others that might want to set up a similar quality and impact assessment activity. These limitations contextualise the data collected and point to the challenges that we encountered. Where possible, we include recommendations to mitigate these challenges.

For data collected directly after training:

- Metrics captured are self-reported, which is subjective and may be biased by various factors such as the respondents’ mood at the time of filling in the survey.
- A quantitative approach is most appropriate to facilitate ease of analysis however there are limitations to the detail that may be captured from quantitative responses. Including an ‘other’ or ‘comment box’ option for each question ensures that free text responses may still be collected that could be analysed by qualitative methods at a later stage thereby enriching the feedback collected. Pilot work to develop a framework to assess qualitative data of this nature is being carried out by ELIXIR Portugal.
- In order to accommodate the various training providers (22 ELIXIR Nodes each comprising many research institutions), demographic information is captured via one of two potential sources, depending on the ELIXIR Node - registration form or feedback survey. As feedback survey responses are not compulsory, while a registration form is, the rates of completion from these two sources is likely different. Where possible, we suggest specifying a single route for data collection so that the same assumptions may be made across the entire dataset. In addition, for those collecting demographic information via registration form, it is worth ensuring that any no-shows are removed from this dataset so as not to skew demographic data.
- Only a subset of participants will fill in the feedback form. However, meaningful conclusions may still be drawn from trends in the data. One might increase the survey response rate by demarcating 10-15 minutes at the end of the training event for participants to fill in the survey.

In addition to the above, for data collected in the longer term, 6 months to one/two years after training:

- Only a subset of participants can be approached to provide feedback in order to comply with GDPR; one may only contact those the provided consent to be contacted. In order to increase one’s mailing list of individuals that may be contacted, we suggest including a question in the survey send out directly after the training event asking for consent to contact the participant at a later stage for further feedback.
- Email contacts might change so one might not be able to approach all individuals who consent.
- The timescale of longer-term feedback collection is often constrained by the time and resources available. We recommend planning for longer term feedback during a project planning/grant writing phase of a project so that realistic timescales may be achieved.
