# Supplementary material for: A framework to assess the quality and impact of bioinformatics training across ELIXIR
Source: PLoS Comput Biol. 2020 Jul 23;16(7):e1007976. doi: 10.1371/journal.pcbi.1007976 (PMC7377377; doi:10.1371/journal.pcbi.1007976)
Supplement: S4 File — (DOCX) [file pcbi.1007976.s004.docx]

S4 File, Long term feedback data supplementary figures.

For S4A - S4C Fig, each respondent was able to select multiple answers to this question. The ‘upset diagram’ illustrates how participants felt that the training has helped them with their work (‘Response Per Option (set size)’, lower LHS) as well as the different combinations of answers selected by the same individual, represented by the size of the intersection (‘Intersection Size’, RHS, ‘orange’). Packages used to create this graph in the R environment: tidyverse, UpsetR.

For S4D - S4G Fig, each respondent was able to select multiple answers to this question. The ‘upset diagram’ illustrates what outcomes the training has led to or facilitated (‘Response Per Option (set size)’, lower LHS) as well as the different combinations of answers selected by the same individual, represented by the size of the intersection (‘Intersection Size’, RHS, ‘orange’).


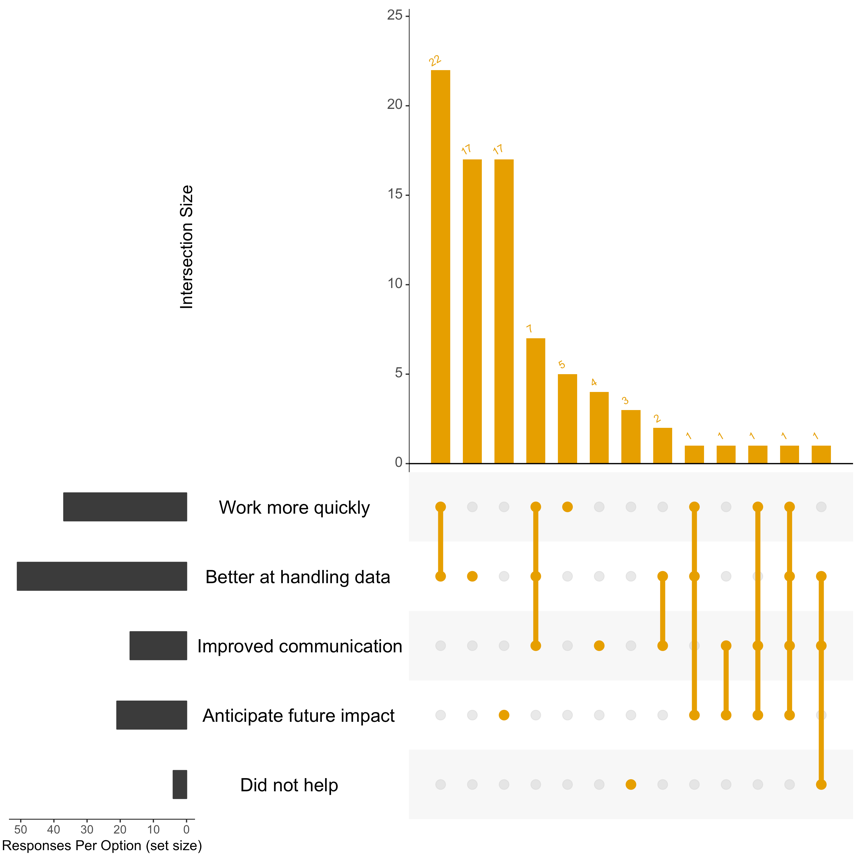


**Fig S4A. How did the training help with your work (6 months after training)?**

**
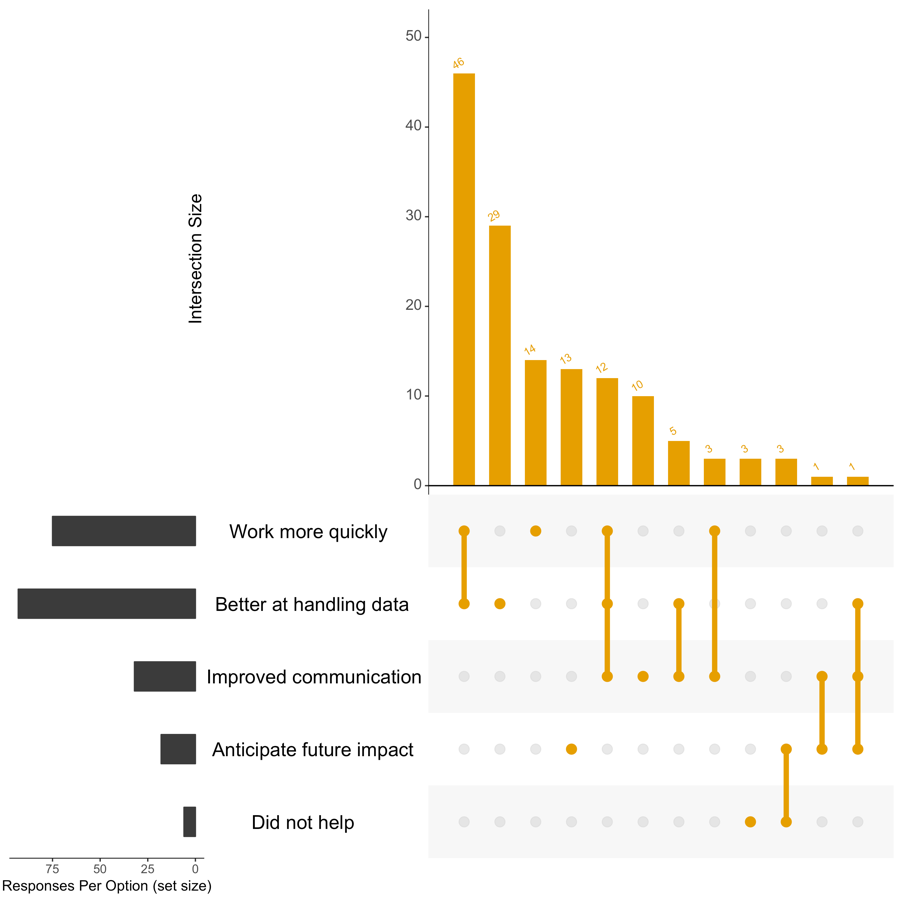
**

**Fig S4B. How did the training help with your work (6 months to 1 year after training)?**


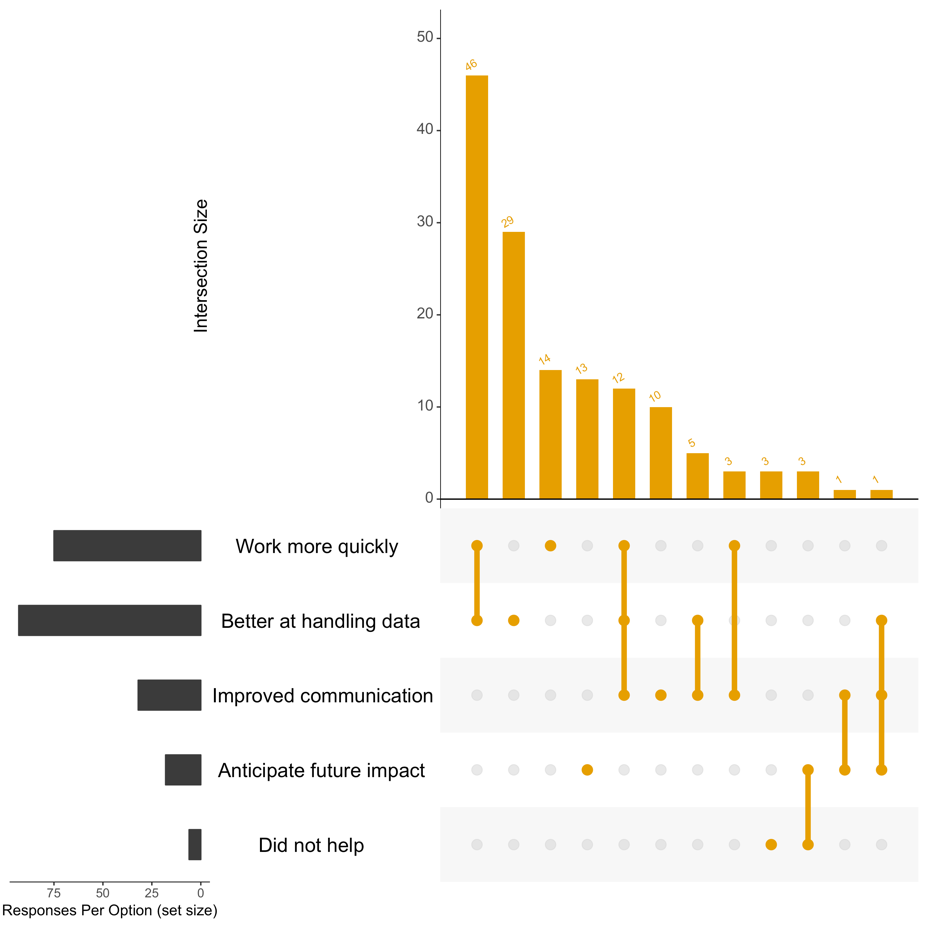


**Fig S4C. How did the training help with your work (more than 1 year after training)?**

**
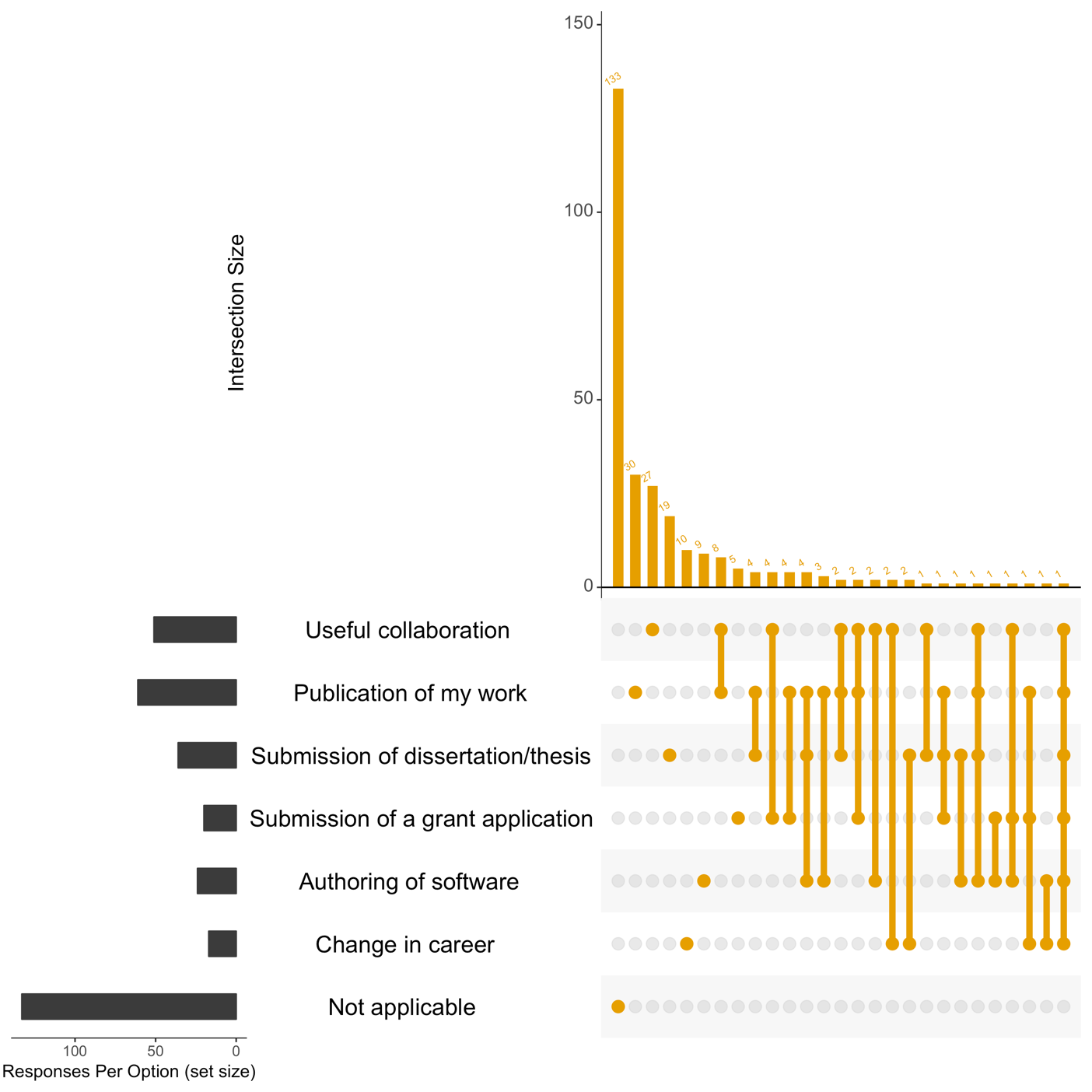
**

**Fig S4D Did the training lead to or facilitate any of the following outcomes (all time points, including not applicable values)?**

**
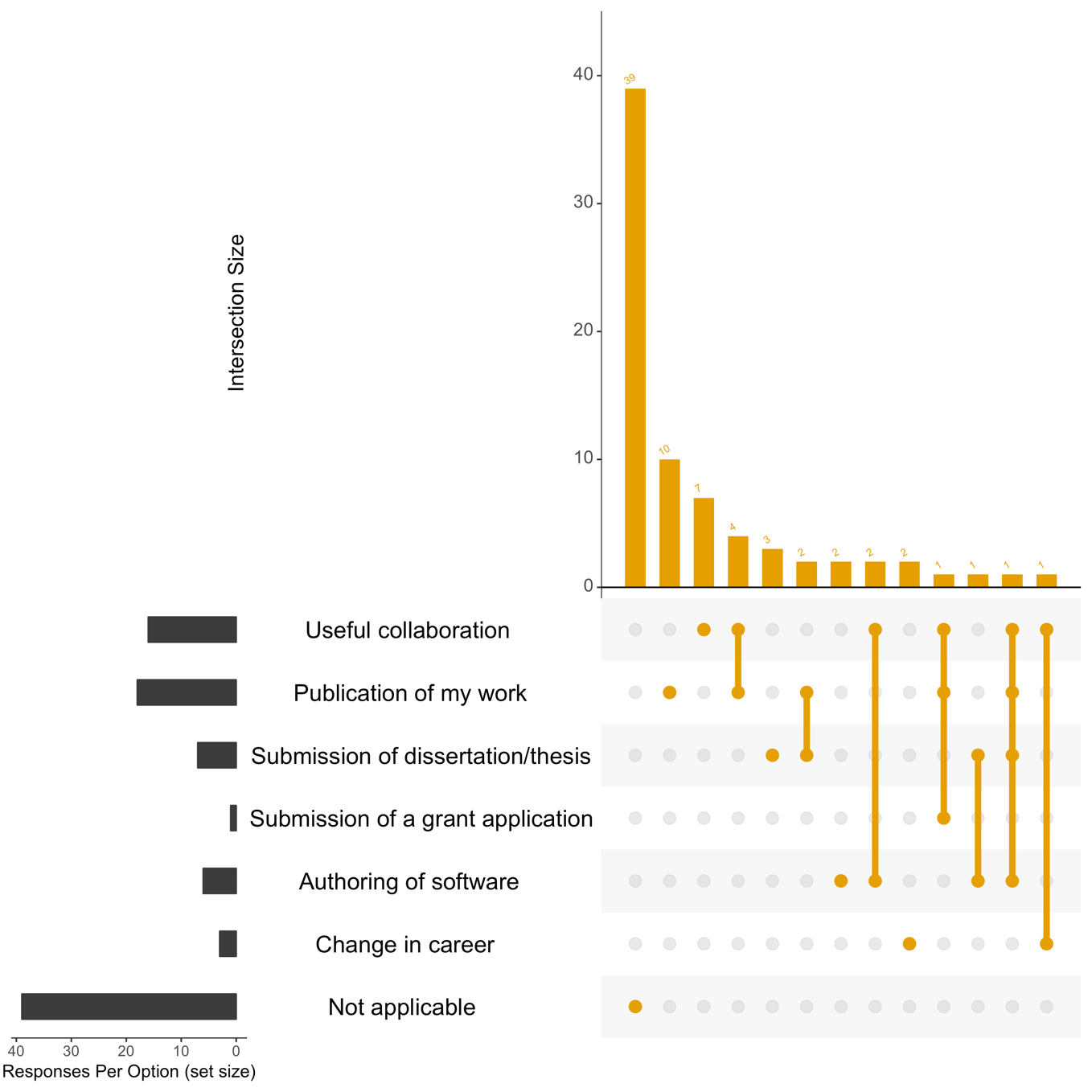
**

**Fig S4E. Did the training lead to or facilitate any of the following outcomes (6 months after training)?**

**
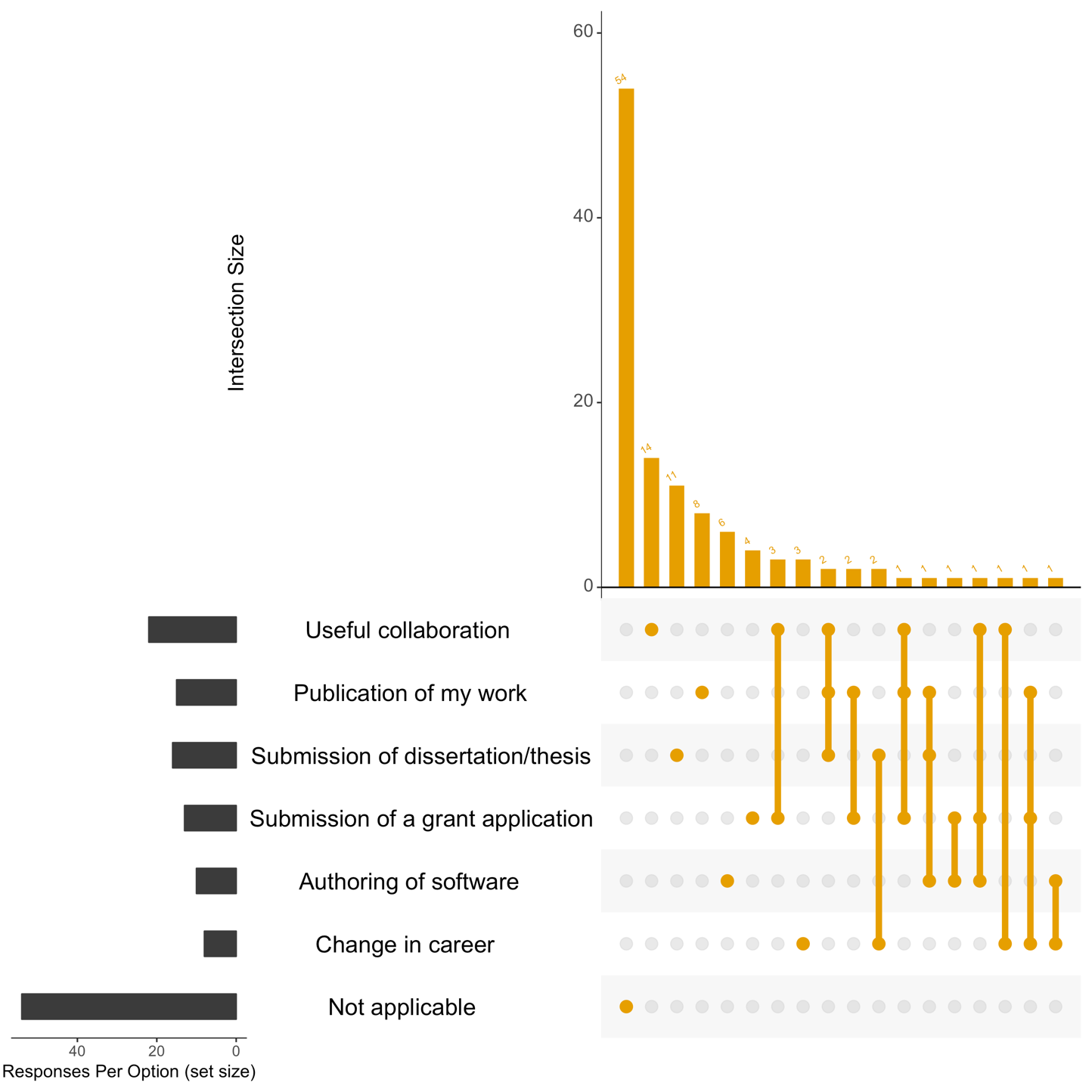
**

**Fig S4F. Did the training lead to or facilitate any of the following outcomes (6 months to 1 year after training)?**

**
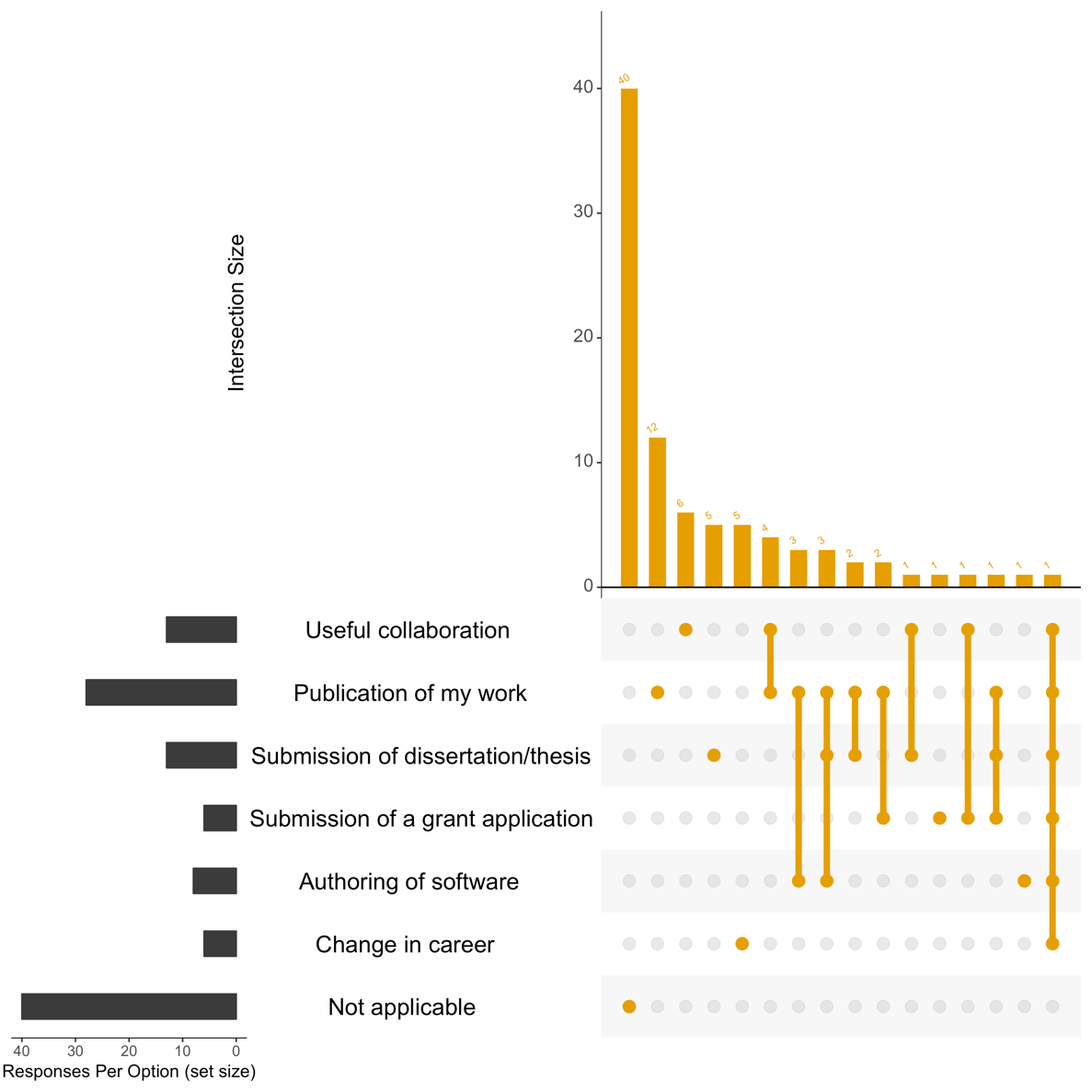
**

**Fig S4G. Did the training lead to or facilitate any of the following outcomes (more than 1 year after training)?**
